# Suicide attempt during ramelteon addition to flunitrazepam: A case report

**DOI:** 10.1002/pcn5.168

**Published:** 2024-01-18

**Authors:** Takeshi Terao

**Affiliations:** ^1^ Department of Neuropsychiatry Oita University Faculty of Medicine Yufu Oita Japan; ^2^ Matsuzaki Clinic Nakatsu Oita Japan

**Keywords:** flunitrazepam, primary insomnia, ramelteon, suicide attempt

## Abstract

**Background:**

Benzodiazepines, such as flunitrazepam, may be at risk of disinhibition, leading to aggressiveness, impulsivity, self‐harm, and possibly suicide attempts, whereas ramelteon may be far from disinhibition.

**Case Presentation:**

In a 43‐year‐old female with primary insomnia, flunitrazepam alone did not induce any type of disinhibition, but the addition of ramelteon to flunitrazepam brought about disinhibition, leading to aggressiveness and finally to her suicide attempt. Her disinhibition rapidly subsided as soon as ramelteon alone was discontinued.

**Conclusion:**

These findings suggest that a suicide attempt may occur during ramelteon and flunitrazepam combination in a susceptible patient.

## BACKGROUND

According to the review by Mandrioli et al.,^1^ ramelteon targets melatonin (MT) 1 and MT2 receptors in the brain. Ramelteon is rapidly absorbed, with peak serum concentrations occurring <1 h after administration, and undergoes extensive first‐pass metabolism. In the liver, ramelteon metabolism is catalyzed by CYP1A2, CYP2C19, and CYP3A4, in decreasing order of importance, whereas only CYP3A4 seems to contribute in the intestine. The apparent elimination half‐life of ramelteon is in the range of 1–2 h, while that of the main metabolite is 2–4 h. The adverse effects were headache, somnolence, fatigue, nausea, dizziness, and insomnia, with an overall incidence similar to that of the placebo. Therefore, ramelteon appears much safer than benzodiazepines, which have various side effects including disinhibition, leading to aggressiveness, impulsivity, self‐harm, and possibly suicide attempt.

In this case report, we present a case of a suicide attempt during ramelteon addition to flunitrazepam. Informed consent was received from the patient to publish this case report.

## CASE PRESENTATION

A 43‐year‐old female presented with primary insomnia and had no psychiatric history. She had no psychiatric comorbidities. Notably, she had a family history of her mother's suicide. She had no psychiatric information about her mother because her mother completed suicide when she was a child. She continued to work at school well. In December 2019, I prescribed her 2 mg/day of flunitrazepam, which resulted in good sleep. Due to her concern about dependence on flunitrazepam, it was gradually decreased to 1 mg/day in September 2020, after which she could sleep. Thereafter, 5 mg/day of lemborexant was administered to change the flunitrazepam without success, therefore she continued taking 1 mg/day of flunitrazepam. In March 2023, flunitrazepam was increased to 2 mg/day owing to the exacerbation of insomnia. In May 2023, a famous Japanese actor attempted suicide with his parents' deaths using a large amount of flunitrazepam, which was broadcast on television. She knew the news and was reluctant to continue taking flunitrazepam. In July 2023, I heard her complaint and added 8 mg/day ramelteon to 2 mg/day flunitrazepam. If ramelteon was effective for her, I planned to gradually decrease flunitrazepam and eventually discontinue it.

After adding ramelteon (8 mg/day) to flunitrazepam (2 mg/day), she was in a daze state, and her brain did not work well during the daytime, while she had a light sleep at night. Seven days after adding ramelteon, she became aggressive and had a violent quarrel with her boyfriend. Nine days after adding ramelteon, she had serious suicidal ideation, pondering how she would complete suicide and whether her family would receive a death benefit. When her boyfriend came to her rescue, she had a narrow escape. Eleven days after adding ramelteon, she consulted a pharmacist, who told her to discontinue 8 mg/day of ramelteon, but to continue 2 mg/day of flunitrazepam. She followed the instruction. Within a few days, her unstable state, including aggressiveness and suicidal ideation, completely subsided and she recovered as before. Six months after ramelteon discontinuation, the patient continued to be well treated with 2 mg/day of flunitrazepam.

## DISCUSSION

In the present case, flunitrazepam alone did not induce any type of disinhibition, but the addition of ramelteon to flunitrazepam brought about disinhibition, leading to aggressiveness, and finally to suicide attempt. Her disinhibition rapidly subsided as soon as ramelteon alone was discontinued, suggesting that ramelteon itself or the ramelteon and flunitrazepam combination induced such serious side effects.

As afore‐mentioned, ramelteon itself appears much safer than benzodiazepines, but in this case ramelteon itself might have induced suicide attempt. As for drug interactions with ramelteon, fluvoxamine can increase the maximum concentration of ramelteon by more than 70 times owing to CYP1A2 inhibition; however, although less intense, the effects can be caused by the simultaneous administration of other CYP subtype inhibitors, such as fluconazole (CYP2C9), ketoconazole (CYP3A4), fluoxetine (CYP2D6), or teophylline, a CYP1A2 substrate that competes with ramelteon.^2^ In contrast, flunitrazepam is mainly metabolized by CYP3A4,^2^ therefore flunitrazepam and ramelteon might have been competitively metabolized by CYP3A4 with elevated levels of flunitrazepam and ramelteon, leading to aggressiveness and finally to her suicide attempt, although this is highly speculative and her suicide attempt could have been just a coincidental one.

The limitations of this case report are the lack of measurement of flunitrazepam and ramelteon levels, and measurement of enzyme polymorphisms for these drugs, particularly CYP3A4.

## CONCLUSION

The present case suggests that ramelteon addition to flunitrazepam may lead to suicide attempt in a vulnerable patient with a family history of suicide.

## AUTHOR CONTRIBUTIONS

This case report was written by Takeshi Terao.

## CONFLICT OF INTEREST STATEMENT

The author declares no competing financial interests or personal relationships that could have appeared to influence the work reported in this paper.

## ETHICS APPROVAL STATEMENT

The Oita University Ethics Committee permits the publication of case report if the patient gives an informed consent.

## PATIENT CONSENT STATEMENT

Written consent to publish this case report was obtained from the patient.

## CLINICAL TRIAL REGISTRATION

N/A.

## Data Availability

N/A.
